# Selective Detection of Fungal and Bacterial Glycans with Galactofuranose (Galf) Residues by Surface-Enhanced Raman Scattering and Machine Learning Methods

**DOI:** 10.3390/ijms26094218

**Published:** 2025-04-29

**Authors:** Julia Yu. Zvyagina, Robert R. Safiullin, Irina A. Boginskaya, Ekaterina A. Slipchenko, Konstantin N. Afanas‘ev, Marina V. Sedova, Vadim B. Krylov, Dmitry V. Yashunsky, Dmitry A. Argunov, Nikolay E. Nifantiev, Ilya A. Ryzhikov, Alexander M. Merzlikin, Andrey N. Lagarkov

**Affiliations:** 1 Institute for Theoretical and Applied Electromagnetics, Russian Academy of Sciences, 125412 Moscow, Russia; jul-zvyagina@inbox.ru (J.Y.Z.); safiullinrk@gmail.com (R.R.S.); slipchenko-katya@mail.ru (E.A.S.); kavacuum@mail.ru (K.N.A.); sedova_marina@mail.ru (M.V.S.); nanocom@yandex.ru (I.A.R.); merzlikin_a@mail.ru (A.M.M.); itae@itae.ru (A.N.L.); 2 N.D. Zelinsky Institute of Organic Chemistry, Russian Academy of Sciences, 119991 Moscow, Russia

**Keywords:** surface-enhanced Raman scattering, machine learning methods, synthetic oligosaccharides, β-D-galactofuranose, principal component analysis, logistic regression

## Abstract

Specific monosaccharide residue, β-D-galactofuranose (Galf) featuring a five-membered ring structure, is found in the glycans of fungi and bacteria, but is normally absent in healthy mammals and humans. In this study, synthetic oligosaccharides mimicking bacterial and fungal glycans were investigated by SERS (Surface-Enhanced Raman Scattering) techniques for the first time to distinguish between different types of glycan chains. SERS spectra of oligosaccharides related to fungal α-(1→2)-mannan, β-(1→3)-glucan, β-(1→6)-glucan, galactomannan of *Aspergillus*, galactan I of *Klebsiella pneumoniae*, and diheteroglycan of *Enterococcus faecalis* were measured. To analyze the spectra, a number of machine learning methods were used that complemented each other: principal component analysis (PCA), confidence interval estimation (CIE), and logistic regression with L1 regularization. Each of the methods has shown own effectiveness in analyzing spectra. Namely, PCA allows the visualization of the divergence of spectra in the principal component space, CIE visualizes the degree of overlap of spectra through confidence interval analysis, and logistic regression allows researchers to build a model for determining the belonging of the analyte to a given class of carbohydrate structures. Additionally, the methods complement each other, allowing the determination of important features representing the main differences in the spectra containing and not containing Galf residue. The developed mathematical models enabled the reliable identification of Galf residues within glycan compositions. Given the high sensitivity of SERS, this spectroscopic technique serves as a promising basis for developing diagnostic test systems aimed at detecting biomarkers of fungal and bacterial infections.

## 1. Introduction

Carbohydrates are one of the major classes of biomolecules, and play a crucial role in cell recognition processes, including the interaction between the host and invading microorganisms. Carbohydrate structures are the “fingerprint” display on the surface of pathogenic fungi among other microorganisms [1,2]. In recent years, polysaccharide structures have been identified as markers for the diagnosis of fungal diseases [3]. In addition to the general fungal marker β-D-glucan [4], there are several specific polysaccharides associated with fungal species. These include mannan for candidoses [5], galactomannan for aspergilloses [6], and mannoxylogalactan for cryptococcosis [7]. A distinguishing characteristic of numerous fungal polysaccharides is the incorporation of galactofuranose residues (Galf), which occur in diverse structural contexts and exhibit variable glycosylation patterns. For example, the most well-characterized polysaccharide marker of *Aspergillus* species is galactomannan (GM), which serves as an established diagnostic standard for invasive aspergillosis in clinical laboratory settings [8]. GM is a highly branched heteropolysaccharide composed of a mannan backbone with side chains built up from β-(1→5)-linked d-galactofuranoside residues. Furthermore, in *Cryptococcus neoformans*, galactofuranoside units are components of the capsular polysaccharide glucuronoxylomannogalactan, where Galf residues are attached to the O-2 and O-3 of the α-(1→6)-linked galactopyranose backbone [9]. Single galactofuranose residues attached to the fungal mannan have also been identified in *Trichophyton mentagrophytes*, *Trichophyton rubrum* [10] and *Paracoccidioides brasiliensis* [11]. Oligogalactofuranoside sequences with β-(1→6) linkages have been characterized as constituents of polysaccharides in *Malassezia furfur* and *Malassezia pachydermatis* [12].

Existing diagnostic methods are not completely effective for the timely detection of fungal infection at an early stage, which is vital for the effective treatment of severe fungal disease associated with high mortality [13]. Currently, the challenge of detecting low concentrations of related carbohydrate markers in the physiological fluids is relevant. This work aims to develop accurate, rapid, and widely applicable protocols for effective fungal glycan diagnostics using highly sensitive spectroscopic methods. Recently, promising results have been shown in the detection of trehalose, a metabolite of *Candida*, in human serum with the appliance of matrix-assisted laser desorption/ionization time-of-flight mass spectrometry (MALDI TOF MS) method. However, the developments in this area have not yet been widely implemented to clinical diagnostics of fungal diseases due to the need for further large-scale prospective studies [14,15,16]. Besides MS, nuclear magnetic resonance (NMR) is a commonly used spectroscopic method for the study of oligosaccharides [17,18]. The method also has prospects for mycosis diagnostics, as demonstrated by the example of the T2Candida Panel [19,20]. However, both MS and NMR are characterized by a high cost of equipment, labor intensity, as well as other drawbacks. In addition, important modern methods for diagnosing fungal infections are colorimetric methods such as ELISA. There are a number of modern works on this topic [21,22,23], allowing the quantitative determination of the content of the infectious marker in the body. However, the results of colorimetric methods are often discrete values, reflecting only the quantitative content of the marker, but not its status. In this context, the possibility of obtaining additional spectral information during the analysis will expand the diagnostic capabilities of the methods.

Surface-enhanced Raman scattering (SERS) has successfully proven itself a highly sensitive method used to enhance Raman spectra and allows the detection of small amounts of biologically significant analytes: proteins, amino acids, viral particles [24,25,26,27,28,29,30,31], and complex living objects, like fungi and bacteria [32,33,34,35,36]. To implement it, specialized nanostructured substrates are used, usually based on gold and silver. Such substrates are obtained by various methods: thermal [37,38], electron-beam [39], and magnetron [40] metal sputtering in a vacuum; colloid nanoparticle synthesis [41]; controlled electro-chemical surface roughening [42,43]; and another methods, including the formation of nano-objects using plasma-chemical etching [44]. The choice of a suitable SERS substrate may be determined by the chemical nature of the analyte and the method of solving its detection problem. For example, in a number of studies, gold [45] and silver [46] nanoparticles were used as SERS-active structures for the identification of fungal infections. In particular, the problem of measuring the spectra of biologically significant protein analytes and enzymes can be solved using nanostructured silver-based substrates formed by electron-beam evaporation in a vacuum [47].

Along with the selection of the optimal SERS substrate, the problem of high-quality and fast processing of spectral information should be solved. This will expand the areas of application of SERS spectroscopy. This task requires the use of modern methods of mathematical data processing and machine learning methods [48,49]. Pre-processing of spectra, methods of reducing dimensionality using the principal component analysis (PCA), linear discriminant analysis (LDA), partial least squares (PLS), etc., as well as the use of classification methods, in particular, logistic regression, allows us to increase the accuracy and reliability of the results of spectral information analysis [50,51]. In particular, PCA is used to visualize differences between classes of spectra and dimensionality reduction [52], and logistic regression (LR) with support vector machine (SVM) allows for efficient classification of spectra based on selected features [53]. In a number of works using SERS as an analytical method, using PCA, LDA, and LR, the problems of quantitative determination of glycated albumin have already been solved [54], the source of angiotensin-converting enzyme has been determined [55], and the SERS spectra of viruses have been obtained and analyzed [56,57].

Vibrational spectroscopy has been used to study carbohydrates for decades. For example, in the work [58], O-H- and C-H-related vibrational modes for D-glucose, maltose, cellobiose, and dextran were analyzed. Laser-Raman spectra of D-ribose and 2-deoxy-D-erythro-pentose in aqueous solution are reported in [59], and the spectral differences between the two sugars are discussed in terms of the structural difference. In study [60], a series of parent monosaccharides and their selectively deuterated derivatives were investigated: α-glucose, α-glucose-1-d, α-glucose-6,6-d2, α-glucose-O-d5, β-glucose, β-glucose-6,6-d2, β-glucose-O-d5, α-galactose, β-galactose, α-mannose and β-mannose and a modified valence force field has been developed to give a least squares agreement between the observed and calculated frequencies for the various isotopic glucoses. This field also gives a satisfactory prediction of the mannose and galactose spectra. A wide range of carbohydrates have been studied by Raman and IR spectroscopy in [61]: monosaccharides (D-(−)-ribose, 2-deoxy-D-ribose, L-(−)-arabinose, D-(+)-xylose, D-(+)-glucose, D-(+)-galactose, D-(−)-fructose) and disaccharides (D-(+)-sucrose, D-(+)-maltose, D-(+)-lactose), and then more complex ones, i.e., trisaccharides (D-(+)-raffinose) and polysaccharides (amylopectin, amylose, glycogen). After this, their spectra were analyzed and the vibration bands were compared. These compounds are main carbohydrate building blocks that play an important role in glycobiology, biochemistry of plant and animal cells, agriculture, food chemistry and many other disciplines. Disaccharides D-maltose, D-maltose-O-d8, D-cellobiose, D-isomaltose, D-gentiobiose, D-trehalose, and α-cyclodextrin were investigated in [62] to determine the stoichiometry of their solution. Some polysaccharides have been studied in [63] to determine their structural characteristics. In [64], Raman optical activity and Raman spectroscopy of the carbohydrates D-(−)-ribose, 2-deoxy-D-(−)ribose, D-(+)-xylose, L-(+)-arabinose, D-(−)-fructose, D-(+)-glucose, 2-deoxy-D-(+)-glucose, D-(+)-galactose, D-(+)-maltose, D-(+)-sucrose, D-(+)-lactose, D-(+)-raffinose, glycogen, and γ-cyclodextrin in solution were investigated. Also, aqueous mixtures of D-mannose and D-glucose in different concentrations were studied in [65]. Some oligosaccharides (trehalose, sucrose, maltose, melibiose, lactose, maltotriose, raffinose, and stachyose) were investigated in [66] for tracking the changes in conformation of oligosaccharides, and the constraints imposed by hydrogen bonding with the solvent.

However, it should be noted that these studies did not require low concentrations. One of the current areas of research is the development of methods for determining oligosaccharides in the body in low concentrations that accompany invasions of the human body by fungi and bacteria. The use of Raman spectroscopy for these purposes is presented only in the work [67], where employed for the characterization and comparison of two different classes of exo-polysaccharides including glucans and fructans which are produced by different bacteria. The PCA was successfully applied to analyze the results in this work. However, only high concentrations of substances were analyzed, which is not applicable to the analysis of human diseases, since a more sensitive method is required.

The above-described success of SERS applications for the analysis of biologically significant analytes in low concentrations (proteins, enzymes [54,55,68]) suggests the admissibility of using the method for the registering compounds of the polysaccharide/oligosaccharide class as well. Monosaccharides have already been investigated by SERS in a number of studies. In particular, several works are devoted to the development of methods for the determining glucose levels in physiological fluids to determine diabetes [69,70,71,72]. In [73], a wide range of monosaccharides (arabinose, fructose, galactose, glucose, mannose, and ribose) were investigated with an emphasis on the surface binding ability to provide a better quality SERS analysis, which was achieved by the additional use of mercaptophenylboronic acid (mPBA). SERS was used for the analysis of glucose enantiomers [74]. Oligosacharides maltotetraose (α-D-Glc-(1→4)-α-D-Glc-(1→4)-α-D-Glc-(1→4)-D-Glc) and stachyose hydrate (α-D-Gal-(1→6)-α-D-Gal-(1→6)-α-D-Glc-(1→2)-β-D-Fru) in a really low concentration of 1 mM were studied by the SERS method in the [75], where their SERS spectra were analyzed using numerical methods.

Due to the complexity of oligosaccharide spectra, a combination of spectroscopy and machine learning methods is required for their processing. This approach will not only improve the quality and speed of spectral analysis but also open up prospects for automation. The promise of this approach has been demonstrated in studies of protein and enzyme detection. These findings indicate the potential for broader application across various biologically relevant assays, including carbohydrate analysis. For example, a large number of saccharides were studied using Raman spectroscopy with PCA in [76]. In study [75], oligosaccharides were identified using the SERS method together with the partial least squares method, and a calibration concentration dependence was constructed.

In the presented study, synthetic oligosaccharides related to glycans, which are part of the cell walls of fungi and bacteria, are analyzed for the first time using the SERS method together with machine learning methods. Some of them, containing the Galf residue, are responsible for pathological conditions of the human organism. The paper presents approaches to obtaining stable reproducible SERS spectra of oligosaccharides in a physiologically significant concentration, suitable for processing by machine learning methods. The vibrational bands of the SERS spectra of each sample are compared. To analyze the spectra, we utilize a number of machine learning methods: principal component analysis, confidence interval estimation, and the L1-regularization approach are used together with the logistic regression method to determine the optimal parameters for identifying the contributions of features responsible for the differences in the spectra. The quality of the model is demonstrated using the metrics that define important parameters of the analysis. The metric «accuracy» represents the proportion of correctly classified instances among the total number of instances; «precision» measures how many of the items identified as positive are truly positive; «recall» indicates how many of the true positives the model manages to detect; and «F1-Score» is the harmonic means of precision and recall, offering a assessing model performance, especially useful for uneven class distributions. Sets of vibrational bands responsible for the differences in the spectra of substances containing and not containing the Galf residue are identified.

## 2. Results and Discussion

### 2.1. AFM of the SERS Substrate

**Figure 1:**
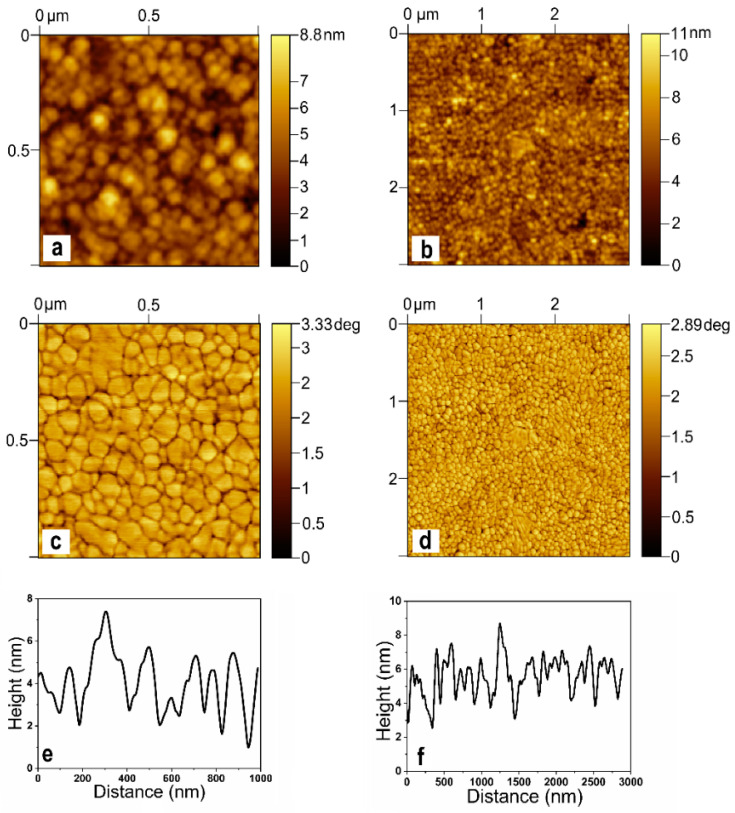
AFM image of the SERS substrate surface areas with dimensions: (**a**) 1 × 1 μm; (**b**) 3 × 3 μm. Phase AFM image of the SERS substrate surface areas with dimensions (**c**) 1 × 1 μm; (**d**) 3 × 3 μm; cross-section on the corresponding surface area (**e**) 1 × 1 μm; (**f**) 3 × 3 μm.

The substrate surface AFM investigation results are presented in Figure 1.

The polycrystalline microstructure is typical of metal films formed by electron beam deposition [77]. From the presented surface profiles (Figure 1e,f), it can be seen that the surface can be described in terms of nanoscale inhomogeneities. Based on the AFM data, the roughness parameters were calculated according to ISO 21920-2:2021 [78]: root mean square roughness (R_q_ = 1.2 ± 0.2 nm); average third-highest peak to third-lowest valley height (R_3z_ = 4.3 ± 0.8 nm); kurtosis (R_ku_ = 3.5 ± 0.9); and average wavelength of the profile (λ_a_ = 183.8 ± 25.0 nm). Parameters R_q_ and λ_a_ indicate the smoothness of the substrate on a macro scale. The presence of local heterogeneity on the submicron scale is characterized by the parameters R_3z_ and R_ku_. The presence of local heterogeneity on the submicron scale is characterized by the parameters R_3z_ and R_ku_. Also, the surface profiles in Figure 1e,f demonstrate that the substrate used in the work has a complex surface morphology with numerous nanoscale depressions and elevations. The observed surface heterogeneities can contribute to the localization of the electromagnetic field, due to which the SERS effect is ultimately realized [79]. The mechanism of SERS implementation on such substrates is described in more detail in [80,81].

### 2.2. Analysis of the Main Vibration Bands of the Analyte Spectra

**Figure 2:**
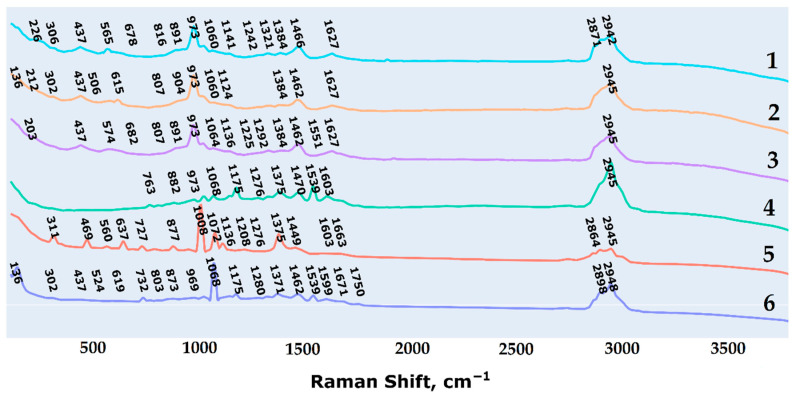
The averaged and normalized spectra of studied analytes.Different colors denote the investigated oligosaccharides related to α-(1→2)-mannan (**1**); β-(1→3)-glucan (**2**); β-(1→6)-glucan (**3**); diheteroglycan of *Enterococcus faecalis* (**4**); galactan I of *Klebsiella pneomoniae* (**5**); galactomannan of *Aspergillus* (**6**).

The averaged and normalized spectra of all analytes are shown in Figure 2.

The main vibration bands of the spectra were assigned on the basis of literature data and are presented in Table 1.

### 2.3. Mathematica Treatment Results

#### 2.3.1. PCA

**Figure 3:**
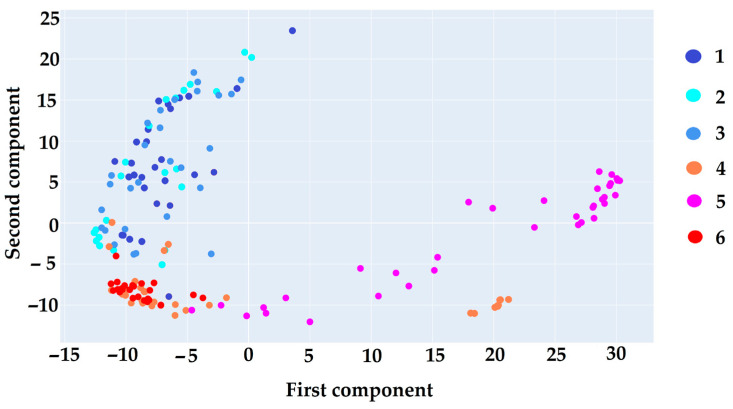
Sample of spectra in projection on the PCA axis. Different colors denote the investigated oligosaccharides related to α-(1→2)-mannan (**1**); β-(1→3)-glucan (**2**); β-(1→6)-glucan (**3**); diheteroglycan of *Enterococcus faecalis* (**4**); galactan I of *Klebsiella pneomoniae* (**5**); galactomannan of *Aspergillus* (**6**).

Figure 3 illustrates the results of processing Raman spectra using PCA, which involved dimensionality reduction and projection of the data onto the principal component axes. This visualization shows how the original high-dimensional spectral data are transformed into a lower-dimensional space, enabling the identification of primary directions of variation that distinguish different analyte groups. The projections onto the principal components highlight the distinct spectral patterns and clusters, thus facilitating the comparison of spectral features and enhancing interpretability by focusing on the most informative variations within the data.

The results displayed in Figure 3 highlight a clear differentiation among oligosaccharide spectra, organizing them into two distinct clusters. The first cluster comprises oligosaccharides without the Galf residue (**1**–**3**), while the second cluster consists of oligosaccharides containing the Galf residue (**4**–**6**). This pattern underscores the substantial spectral differences between the two groups and supports the feasibility of accurate classification through machine learning approaches, particularly the classification via LR. The observed distinctions emphasize the Galf residue impact on spectral characteristics, positioning it as a key feature to enhance the precision of classification models.

#### 2.3.2. Determination of the Vibration Bands Responsible for the Separation of Spectra

**Figure 4:**
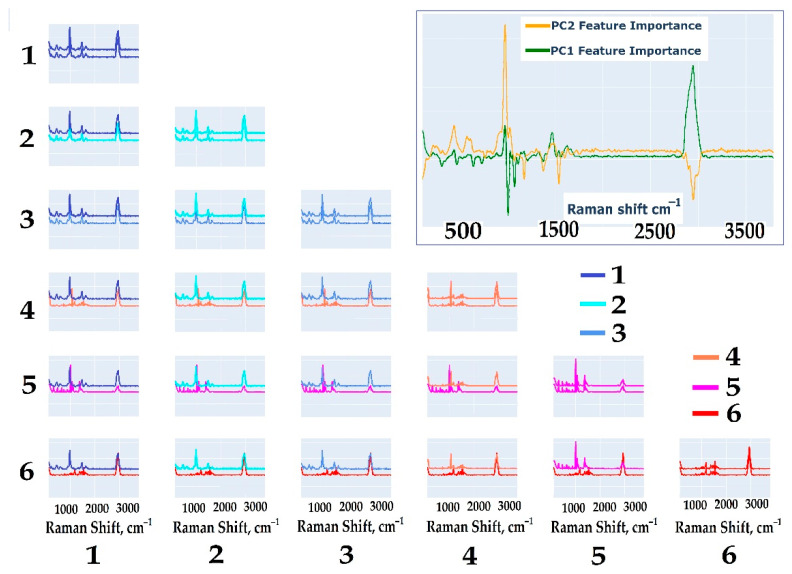
Comparison of variance captured in principal components for each pair of analytes. The inset shows the loading spectra for the first and second components calculated for the averaged spectra of residue-free Galf oligosaccharides and residue-containing Galf oligosaccharides.

The PCA method enables the identification of loadings that reflect the contributions of different spectral regions to the overall spectral variance corresponding to specific groups. Chemically, these regions can be interpreted as vibrational bands responsible for distinguishing spectra. Figure 4 presents the PCA results applied to the spectra of all studied oligosaccharides. Each subplot displays spectra from two analytes and highlights the contribution of the first two principal components (PC1 and PC2) to the differences between these spectra. The general variation directions of the sample align with the variations observed within each pair of analyte spectra, allowing for the identification of key spectral regions where differences between the two substances are most pronounced. Notably, most of the variance corresponds to peaks present in one analyte’s spectrum but absent in the others. These distinguishing features are highly informative for class separation, as evidenced by the contributions of the first two principal components.

By analyzing patterns that emerge when comparing rows or columns within the pairwise comparison matrix, the following key characteristics become apparent:

- Oligosaccharide groups without the Galf residue (**1**–**3**) exhibit similar spectral profiles. In pairwise comparisons, the differences between these groups are minimal and centered around shared peaks, indicating the presence of stable and comparable spectral fragments.
- Oligosaccharide groups containing the Galf residue (**4**–**6**) display distinct spectral differences. Their vibrational bands vary significantly when compared to those of oligosaccharide groups without Galf, indicating the strong influence of Galf on the spectral profile. This distinctive spectral behavior allows these groups to be categorized separately, highlighting their unique properties.

The identified patterns provide a deeper understanding of the nature of differences between classes, justifying the use of PCA and selected spectral features for further development of classification models.

Figure 4 reveals that spectral differences are present across each pairwise comparison. These differences are reflected in the loadings spectra as peaks and troughs, specifically at bands 969, 973, 978, 995, 999, 1004, 1008, 1012, 1017, 1060, 1064, 1068, 1072, 1077, 1081, 1367, 1371, 1375, 1379, 2860, 2864, 2867, 2871, 2874, 2877, 2881, 2884, 2888, 2891, 2894, 2898, 2901, 2904, 2908, 2911, 2915, 2918, 2921, 2925, 2928, 2931, 2935, 2938, 2942, 2945, 2948, 2952, 2955, 2958, 2962, 2965, 2968, 2972, 2975, 2979, 2982, 2985, 2989, 2992, and 2995 cm^−1^.

These vibration bands will be further refined in this study using an alternative calculation method that considers the requirements for the physicochemical interpretability of the vibration bands.

#### 2.3.3. Confidence Interval Estimation

**Figure 5:**
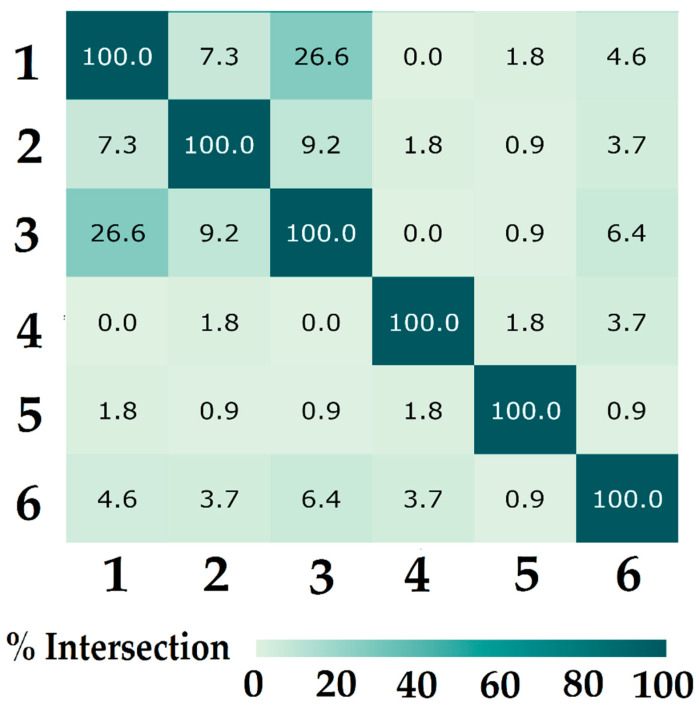
Comparison matrix of the examined samples. The values in each cell represent the percentage of statistically significant overlap for the confidence intervals across all analyzed peaks for each pair of samples.

To confirm the differences in analyte spectra, a method for estimating 95% confidence intervals of intensities at selected vibrational bands was applied using bootstrapping. This approach examined all possible variations in analyte comparisons at the bands identified through PCA, corresponding to intensity peaks. The results, which highlight those bands where statistically significant differences are present, are shown in Figure 4 and Figure 5.

Figure 5 presents a heatmap illustrating the percentage overlap of confidence intervals across key vibrational bands for each pair of oligosaccharide samples. Samples without Galf residue show higher overlap percentages, reflecting greater similarity in their spectral profiles. In contrast, samples with Galf residue exhibit lower overlap values, emphasizing more distinct differences in their vibrational bands and structural features. This reduced overlap highlights the unique spectral characteristics introduced by the presence of the Galf residue.

The vibrational bands with no overlap in confidence intervals between analyte pairs are listed in Table 2.

The primary vibrational bands distinguishing oligosaccharides without Galf residues from those containing Galf are located at 140, 311, 468, 637, 1012, 1016, 1068, 1110, 1072, 1174, 1375, 1461, 1469, 1538, 2894, 2941 and 2944 cm^−1^.

On the other hand, oligosaccharides with Galf residues (samples **4**–**6**) exhibit significantly lower overlap levels with non-Galf oligosaccharides and among themselves, indicating substantial structural differences. This distinct separation underscores the potential for developing a computational model to classify these groups effectively, a process which is further explored in the subsequent sections.

#### 2.3.4. Computational Experiment

In the computational part of our study, we focused on using LR with L1 regularization to classify oligosaccharides into two distinct groups: those containing the Galf residue and those without. The goal was to fine-tune model parameters to maximize classification accuracy while minimizing the number of selected features, thus isolating the most informative spectral bands and producing a more robust model.

To achieve this, we employed a cross-validation technique to optimize the regularization parameter C, which controls the strength of regularization. Smaller C values apply stronger regularization, favoring sparsity, while larger values reduce this constraint. Parameter exploration ranged widely, from 10^−6^ to 10^6^, ensuring thorough investigation of the regularization effects on model performance.

For each value of C, the following steps were carried out:

- Training the logistic regression model on a training subset;
- Assessing classification accuracy on the test subset;
- Recording the count of non-zero coefficients to measure feature selection;
- Mapping selected features relative to key spectral peaks identified by PCA and confidence interval analysis.

**Figure 6:**
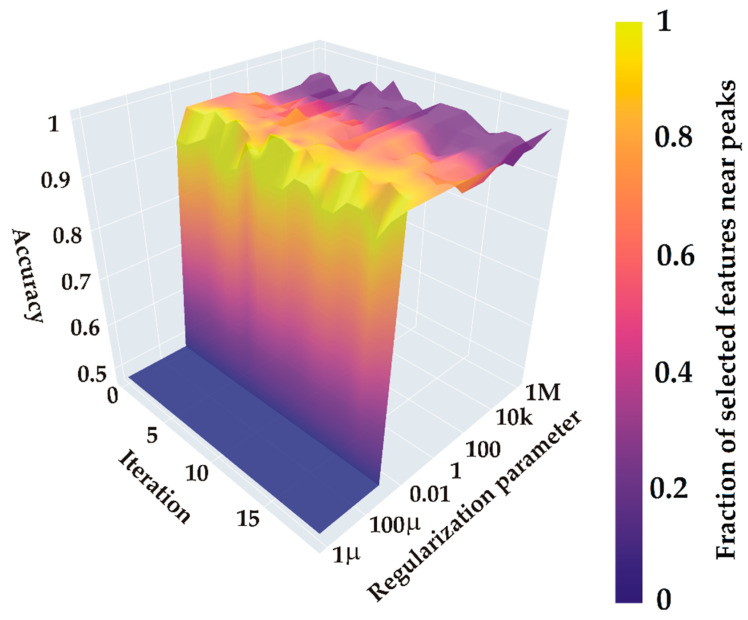
Computational experiment results for identifying the optimal model to distinguish spectral groups based on characteristic peaks.

The results, illustrated in Figure 6, show the regularization coefficient C along the x-axis, while the y-axis displays cross-validation iterations with different data splits. The surface height reflects classification accuracy across C values and iterations, and a color gradient represents the proportion of selected features aligned with previously identified spectral peaks. This visualization highlights the impact of feature selection on model accuracy and the role of spectral characteristics in effective classification.

Figure 6 presents a surface plot that illustrates the relationship between classification accuracy, the regularization parameter C, and training iterations. Our aim was to achieve a balance between classification accuracy and the proportion of selected features, ensuring they align closely with significant spectral bands rather than regions without any signal. This balance is essential for improving model robustness and ensuring that the identified spectral features are consistent across experiments for a given analyte, thereby reinforcing their reliability as classification markers.

The plot demonstrates that model accuracy varies with changes in the regularization parameter C. Our objective was to identify a value of C where selected features predominantly align with spectral peaks, rather than with spectral noise, ensuring solution stability across different training set splits. We observed that a C value of approximately 0.11 provides the optimal balance, yielding high classification accuracy while selecting meaningful features (~100% of the features selected by L1 regularization correspond to significant intensity peaks). This balance reinforces model stability on test data, underscoring the robustness of the approach.

#### 2.3.5. Model Estimation

**Figure 7:**
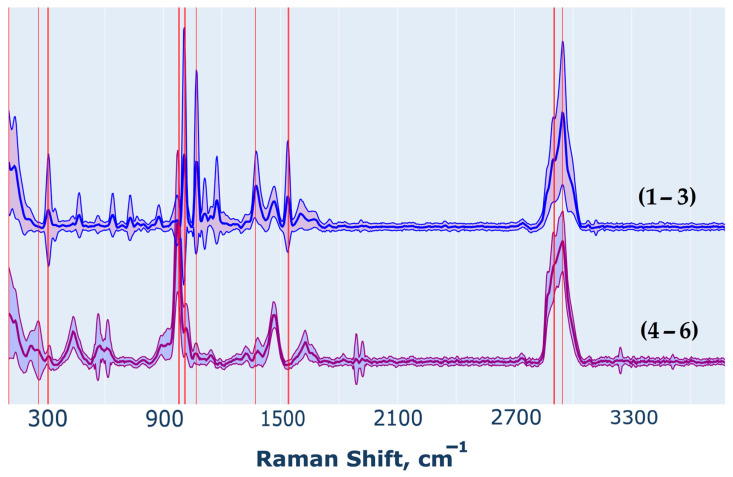
Average spectra after preprocessing for each group, with standard deviation, and the most significant vibrational bands (red vertical line), identified from the computational experiment as key differentiators between oligosaccharides with and without Galf.

Based on the optimized parameters, key features were identified that distinguish the spectra of analytes containing Galf from those without, as illustrated in Figure 7. Using the data of Figure 6, which illustrate the relationship between classification accuracy and the proportion of selected spectral bands, we determined an optimal regularization parameter C that maximizes classification accuracy while isolating features aligned with significant spectral peaks. The identified bands—306, 973, 982, 1007, 1068, 1371, 1539, 2867, and 2945 cm^−1^—demonstrate the model’s robustness in effectively distinguishing between oligosaccharides with and without Galf. While fewer in number than the bands identified through PCA, these selected bands closely correspond in terms of vibrational frequency. This reduction in feature count is a result of refined regularization parameter tuning and an emphasis on maintaining the physical and chemical interpretability of the highlighted vibrational bands.

#### 2.3.6. Classification Results

Using the selected regularization parameter C ≈ 0.11 from the surface plot in Figure 6, an optimal LR model was constructed to classify the two groups: oligosaccharides with and without Galf residue. Table 3 presents the classification metrics, which indicate high accuracy for the resulting model.

According to Table 3, all metrics showed high results. The precision, recall, and F1-score values for both groups are 97%, indicating the model’s high reliability. It is important to note that for the method to be reliable, all metrics must show high results, since it is important to correctly identify both true positive and true negative cases. This is exactly what our result demonstrates. At the same time, the overall accuracy is also high, which shows that the proposed mathematical model effectively distinguishes oligosaccharides with Galf residue from those without it.

## 3. Materials and Methods

### 3.1. Research Objects

**Figure 8:**
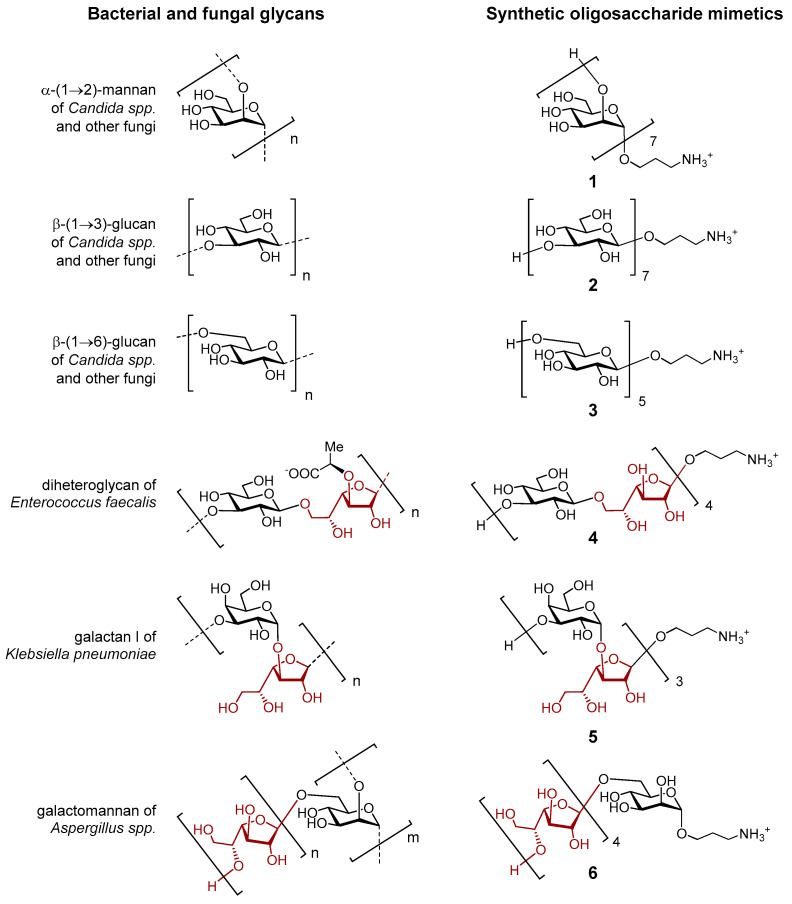
Bacterial and fungal glycans and the corresponding synthetic oligosaccharide mimetics representing the subjects of this study. Galactofuranoside residues in the structure of glycans are highlighted in brown color.

The objects of the study are synthetic oligosaccharides structurally related to bacterial and fungal glycans [82]. Oligosaccharides **1**–**3** (Figure 8) correspond to fragments of fungal α-(1→2)-mannan, β-(1→3)-glucan, and β-(1→6)-glucan, respectively, which are components of the cell wall in pathogenic fungi of the genus *Candida*. These glycans are constructed from monosaccharide residues of only one type: α-mannopyranose for **1**, and β-glucopyranose for **2** and **3**. Oligosaccharides **4**–**6** are heterosaccharides composed of two types of monosaccharide residues, one of which is β-D-galactofuranose (Galf). Oligosaccharides **4**–**6** are fragments of diheteroglycan of *Enterococcus faecalis*, galactan I of *Klebsiella pneumoniae* and galactomannan of *Aspergillus* spp, respectively. The synthesis of compounds **1**–**6** was reported by us previously (**1 [82]**, **2 [83]**, **3 [84]**, **4 [85]**, **5 [86]**, **6 [87,88]**).

### 3.2. SERS Substrate Fabrication

The SERS substrates were prepared according to the technique presented in [88]. The silver films were obtained by electron beam deposition of silver on the glass slides (Minilab, Moscow, Russia) in a URM 3.279.072 vacuum chamber (“Quartz”, Kaliningrad, Russia). The metal sputtering process was carried out under the following conditions: chamber pressure below 6.7 × 10^−4^ Pa, cathode voltage 12 V, beam current 30 mA and accelerating source voltage 8 kV. Before loading into the chamber, the slides were cleaned with isopropyl alcohol (99.5% pure, Sigma-Aldrich, St. Louis, MO, USA), and then after loading they were re-cleaned with plasma in a residual atmosphere at a pressure of 0.133 Pa before the deposition process. High-purity granulated silver (≥99.99%, “Special Alloys Processing Plant”, Moscow, Russia) with a grain size of 3 mm was used as a metal source.

### 3.3. AFM Studies of Substrate Surface

The study of the SERS substrate morphology was performed by atomic force microscopy (AFM) using a Solver Pro microscope (NT-MDT, Zelenograd, Russia) in the tapping mode. The images were analyzed in the Gwyddion 2.60 software (CMI, Brno, Czech Republic). Statistical analysis of the morphology parameters was performed using the built-in Gwyddion software modules, which provide calculation of roughness parameters according to the ISO 21920-2:202 standard.

### 3.4. Analyte Preparation and Application

Solutions of oligosaccharides were prepared in deionized water (Milli-Q, Merck group, Darmstadt, Germany) at a concentration of 100 pg/mL. Aliquots of 3 µL were deposited to SERS substrates using a high-precision microdoser. The drops were dried in air under normal conditions. The SERS spectra of oligosaccharides were measured in the dry precipitate region.

### 3.5. Spectra Measurements

The SERS spectra were measured using a Raman spectrometer Alpha 300R (WITek, Ulm, Germany) based on a confocal microscope with a Zeiss Epiplan Neoflural 50×/0.8 objective. The measurement parameters were as follows: excitation laser wavelength 532 nm, laser power 1.5 mW, accumulation time of one spectrum 0.1 sec, number of spectral averaging 20. A total of 50+ spectra were obtained from each sample.

### 3.6. Pre-Processing of Spectral Data

Spectral data preprocessing is a crucial step to ensure high-quality analysis. In this study, the following operations were applied to the spectra.

#### 3.6.1. Outliers Removing

Outliers were identified and removed using the Isolation Forest method, which builds random trees and uses distance metrics to detect anomalous values. This approach isolates outliers by assessing how readily certain data points can be distinguished from the main dataset. In this study, the contamination parameter was set to 0.1, meaning 10% of data points were deemed outliers and removed accordingly.

#### 3.6.2. Spectra Filtering

To reduce noise levels, the Savitzky–Golay filter was employed, preserving critical spectral features, such as peaks, during the smoothing process. Key parameters included a window size of 9 (where each data point was averaged with neighboring points within a 9-point window) and a polynomial order of 3, defining the polynomial’s degree used for smoothing.

#### 3.6.3. Baseline Correction

To counter baseline fluctuations that may distort spectral data, Asymmetric Least Squares Smoothing (ALS) was utilized. This method adjusts the baseline by eliminating systematic intensity variations unrelated to the analytical signals. Parameters include λ, which determines the extent of baseline suppression, and p, controlling asymmetry to optimize smoothing.

#### 3.6.4. Spectra Normalization

The standard normal variation (SNV) method was applied to account for intensity variations due to different measurement conditions, making spectra more comparable. This normalization involved calculating the mean and standard deviation for each spectrum, reducing systematic deviations and enhancing data uniformity. Each preprocessing step in this pipeline is designed to improve data quality, thus ensuring that subsequent spectral analyses are more accurate and resilient to environmental conditions.

### 3.7. Analyte Discerning Differences Investigation and Their Logistic Regression Classification

This study employed a comprehensive approach to discern differences across the spectral data of various oligosaccharides and improve classification efficacy. By integrating PCA for dimensionality reduction with LR featuring L1 regularization, this method facilitated the extraction of relevant features while enhancing classification accuracy.

#### 3.7.1. PCA

The PCA approach was implemented to reduce the dimensionality of the spectral data and capture the main directions of variance within the dataset. The initial spectra were organized into a matrix, with rows corresponding to individual measurements and columns reflecting intensity values across different Raman shifts. Prior to applying PCA, data centering was conducted to standardize each variable.

By applying PCA across the entire dataset, we identified general patterns that allowed for comparability between different analyte groups. This process highlighted key spectral regions that differentiate oligosaccharides with Galf residues from those without, thereby enhancing interpretability and relevance for subsequent classification tasks.

#### 3.7.2. Estimation of Overlap of Intensity Distributions at Peaks

To compare spectra from different analytes and establish statistical significance in observed differences, we utilized a bootstrapping approach. Bootstrapping is a non-parametric method that estimates the distribution of a statistic, such as the mean, through repeated resampling of the original data with replacement. This technique was selected due to its flexibility and suitability for small sample sizes and non-normally distributed data, which are common characteristics of intensity measurements at specific spectral peaks (vibrational bands). Traditional parametric methods, which depend on assumptions of normality, may not provide reliable confidence intervals under these conditions. In contrast, bootstrapping allowed us to estimate confidence intervals for mean intensities in each group with greater robustness, enabling a more accurate assessment of variability without distributional constraints.

The following section details the application of bootstrapping for calculating confidence intervals and quantifying overlap between groups, providing a foundation for identifying statistically significant differences across analytes.

#### 3.7.3. Bootstrapping Procedure

For each peak p_i_ and each analyte group, the bootstrapping process involves the following:

1. Generating Bootstrap Samples: Random sampling with replacement is conducted on the original intensity measurements at p_i_, producing 10,000 bootstrap samples;
2. Calculating Statistics: The mean intensity is computed for each bootstrap sample;
3. Building the Empirical Distribution: The distribution of mean values forms an empirical distribution of the mean intensity for each group at p_i_;
4. Deriving Confidence Intervals: From the empirical distribution, confidence interval bounds are determined at a specified confidence level (95%).

Using the parameters obtained from the bootstrapped distributions, we calculated the overlap between confidence intervals for each peak by applying the overlap formula for the paired *t*-test [89]:

(1) $$\omega =\frac{\left(t_{\alpha /2,n-1}(S_{x}+S_{y})/(\sqrt{n}-(\bar{X}-\bar{Y}\right)}{\left(t_{\alpha /2,n-1}(S_{x}+S_{y})/(\sqrt{n}+(\bar{X}-\bar{Y}\right)}\times 100\%$$

where:

- t_α/2,n−1_ is the critical *t*—value at α/2 for n − 1 degrees of freedom.
- *S_x_* and *S_y_* are the standard deviations for groups and, respectively.
- $\bar{X}$ and $\bar{Y}$ are the mean values for groups and.
- *n* is the sample size.

This formula defines the α—overlap for paired tests, providing an alternative method to assess statistical distinction based on bootstrapped data.

#### 3.7.4. Statistical Significance Threshold

To determine significant differences based on the overlap percentage, a critical threshold ω_r_ is used, below which the null hypothesis of no difference is rejected. This threshold is calculated by [90]:

(2) $$\omega_{r}=\left(\frac{1+\sqrt{F_{0}}-\sqrt{1+F_{0}-2_{r}\sqrt{F_{0}}}}{1+\sqrt{F_{0}}+\sqrt{1+F_{0}-2_{r}\sqrt{F_{0}}}}\right)\times 100\%$$

where:

- *r* is the correlation coefficient between the standard deviations of intensities for two groups.
- $F_{0}={\left(\frac{S_{x}}{S_{y}}\right)}^{2}$, where *S_x_* and *S_y_* are standard deviations for each group.

This threshold provides a criterion for rejecting the null hypothesis if the overlap is below, allowing us to conclude significant differences between groups with a 5% significance level.

#### 3.7.5. Application of the Method

This analytical method was implemented to detect specific spectral peaks where the bootstrapped confidence intervals of mean intensities indicated statistically significant differences between analyte groups. By leveraging bootstrapping, this approach provides robust statistical power, particularly valuable when working with small sample sizes and data that may not conform to normal distribution assumptions. Consequently, it enabled the identification of peaks in the spectral data that exhibit meaningful inter-group differences, facilitating a reliable comparison even under these challenging conditions.

#### 3.7.6. Class Similarity Assessment

To evaluate the similarity of mean spectral intensities across all peaks, we calculated the number of statistically non-significant differences for each pairwise comparison across relevant spectral peaks. This metric—the proportion of non-significant overlaps—was then used to quantify class similarity. A higher proportion of overlaps suggests a closer resemblance between classes in terms of their Raman spectral signal, serving as an objective measure of class proximity based on spectral characteristics.

#### 3.7.7. LR with L1 Regularization

The LR model was utilized for classifying spectra and selecting the most informative spectral features, with L1 regularization. This method not only predicts the probability of a sample belonging to a particular class but also performs feature selection by penalizing the sum of the absolute values of the coefficients.

The LR model is represented by the following equation:

(3) $$P\left(y=1|x\right)=\frac{1}{1+e^{-(w^{T}x+b)}}$$

where:

x—sample feature vector;
w—vector of weight coefficients;
b—free term.

The loss function with L1 regularization is expressed as:

(4) $$L\left(w,b\right)=-\sum_{i=1}^{n}[y_{i}lnP(y_{i}|x_{i})+(1-y_{i})\ln(1-P(y_{i}|x_{i}))]+\lambda \sum_{j=1}^{p}\left|w_{j}\right|$$

where:

$y_{i}$—class label for i-th sample $\left(y_{i}\in \left\{0,1\right\}\right)$;
λ—regularization coefficient;
p—number of features.

The optimal value for the hyperparameter $C=\frac{1}{\lambda }$ was selected through grid search within a range from 10^−6^ to 10^6^. This approach aimed to balance model accuracy, coefficient sparsity, and solution stability.

#### 3.7.8. Classification Metrics

The performance of the logistic regression model was evaluated using the following metrics:

Accuracy: The overall proportion of correct predictions, providing a general view of model performance. Represents the proportion of correctly classified instances among the total number of instances. A=(TP+TN)/(TP+TN+FP+FN), where: *TN*—truly negative.

Precision: The ratio of true positives to the sum of true and false positives, indicating the model’s accuracy in identifying the target class. Measures how many of the items identified as positive are truly positive. P=TP/(TP+FP), where: *TP*—truly positive; *FP*—false positive.

Recall: The proportion of true positives out of all actual positives, measuring the model’s ability to capture relevant instances of the target class. Indicates how many of the true positives the model manages to detect. R=TP/(TP+FN), where: *FN*—false negative.

F1-Score: The harmonic means of precision and recall, offering a balanced metric for assessing model performance, especially useful for uneven class distributions.

(5) F1=2(Precision×Recall)/(Precision+Recall)

These metrics together provide a comprehensive assessment of the model’s performance, highlighting its specificity, sensitivity, and overall accuracy.

## 4. Conclusions

We have offered an approach based on selective SERS detection and a machine learning definition of fungal and bacterial glycans with galactofuranose (Galf) residues. This study demonstrates for the first time the measurement the SERS spectra of oligosaccharides without (fungal α-(1→2)-mannan, β-(1→3)-glucan, β-(1→6)-glucan) and with Galf residue (galactomannan of *Aspergillus*, galactan I of *Klebsiella pneumoniae*, diheteroglycan of *Enterococcus faecalis*) at a physiologically significant concentration of 100 pg/mL on nanostructured SERS substrates. To solve the task presented in the work, a SERS substrate was used, which has proven itself to be effective for measuring the SERS spectra of oligosaccharides. It was obtained by electron-beam sputtering and is characterized by the following roughness parameters: root-mean-square roughness R_q_ = 1.2 ± 0.2 nm; average third highest peak to third lowest valley height R_3z_ = 4.3 ± 0.8 nm; kurtosis R_ku_ = 3.5 ± 0.9 and average wavelength of the profile λ_a_ = 183.8 ± 25.0 nm. This substrate allowed us to obtain highly stable SERS spectra of the studied oligosaccharides due to optimally selected parameters for measuring the spectra. The work also showed the importance of preprocessing the spectra to obtain the results. Several sequential methods were used for this purpose: outliers were removed using the Isolation Forest method; the Savitzky–Golay filter was employed to reduce noise levels, baseline correction was performed with Asymmetric Least Squares Smoothing and Spectra were normalized by Standard Normal Variation method.

The number of machine learning methods considered in this paper, such as the PCA, LR with linear regularization L1, and the method for assessing the intersections of confidence intervals for average values on certain vibrational bands (bootstrap), demonstrated their applicability for analyzing the array of analyte spectra considered in the article. Applicability of the approach was confirmed by the number of the metrics: precision, recall, and F1-score. They all show high results of 97%, which shows the reliability of the presented method. At the same time, the overall accuracy was also 97%. The PCA was used to determine the contributions of features. The bootstrap method was used to test the possibility of separating analyte classes through assessing the overlap of confidence intervals obtained during multiple sampling of subsamples. The optimal parameters were selected in the regularized LR to obtain chemically interpretable contributions of features representing vibrational bands and responsible for differences in the SERS spectra of oligosaccharides containing the Galf residue. Using the LR method, a table of metrics was formed based on the obtained optimal parameter C to divide analytes into classes with and without Gulf residue.

The conducted research opens the way towards the development of innovative protocols for clinical diagnosis of bacterial and fungal infections. Their rapid detection will allow for the prescription of accurate treatment in a short time, which will lead to a decrease in mortality from a number of diseases, especially fungal and bacterial pneumonia. The proposed mathematical approaches are universal and can be applied for the analysis of other clinically significant analytes.

## Figures and Tables

**Table 1:** Main vibration bands.

Bands, cm^−1^						Band Assignment	Ref.
1	2	3	4	5	6		
	136				136		
		203					
	212						
226							
306	302	-	-	-	302		
-	-	-	-	311	-		
437	437	437	-	-	437	δ(CCO)	[65]
-	-	-	-	469	-	δ(CCO)	[64]
-	506	-	-	-	-		
-	-	-	-	-	524		
565	-	-	-	560			
-	-	574	-	-	-		
-	615	-	-	-	619		
-	-	-	-	637	-		
678	-	682	-	-	-		
				727	732		
-	-	-	763	-	-	δ(COH)	[65]
-	807	807	-		803		
816				-	-		
-	-	-	-	877	873	δ(COH)	[65]
-	-	-	882	-	-		
891	-	891	-		-		
	904	-	-	-	-		
973	973	973	973	-	969	ν(CO)	[65]
				1008			
1060	1060	1064	1068	1072	1068	ω(CH_2_)	[65]
	1124						
1141	-	1136	-	1136	-	δ(COH)	[65]
-	-	-	1175	-	1175		
-		-	-	1208	-		
-	-	1225	-	-	-		
1242	-	-	-	-	-		
-	-	-	1276	1276	1280		
-	-	1292	-	-	-		
1321							
-	-	-	1375	1375	1371	ω(CH_2_)	[66]
1384	1384	1384	-	-	-	ω(CH_2_)	[66]
-	-	-	-	1449	-	δ(CH_2_)	[64]
1466	1462	1462	-	-	1462	δ(CH_2_)	[65]
-	-	-	1470	-	-	δ(CH_2_)	[61]
-		-	1539	-	1539		
-	-	1551	-	-	-		
-	-	-	1603	1603	1599		
1627	1627	1627	-	-	-		
-	-	-	-	1663	1671		
-	-	-	--	-	1750		
2871	-	-	-	2864	-		
-	-	-	-	-	2898	ν(CH)	[61]
2942	2945	2945	2945	2945	2948	ν(CH_2_)	[61]

Vibration types: ν—stretching; δ—bending; ω—wagging; τ—twisting.

**Table 2:** Vibrational bands distinguishing analytes in pairwise comparisons.

Analyte	2	3	4	5	6
**1**	140, 311, 468, 637, 1012, 1016, 1068, 1375, 1461, 1469, 1538, 2894, 2941	140, 311, 468, 637, 1012, 1016, 1110, 1174, 1461, 1469, 1538, 2894, 2941, 2944	140, 311, 468, 637, 1012, 1016, 1068, 1072, 1110, 1174, 1375, 1461, 1469, 1538, 2894, 2941, 2944	140, 311, 468, 637, 1012, 1016, 1068, 1072, 1110, 1174, 1375, 1461, 1469, 1538, 2894, 2941, 2944	1012, 1016, 1068, 1072, 1110, 1174, 1375, 1461, 1469, 1538, 2894, 2941, 2944
**2**		140, 1012, 1016, 1110, 1174, 1375, 1538, 2941, 2944, 1461	140, 311, 468, 637, 1012, 1016, 1068, 1072, 1110, 1174, 1375, 1461, 1538, 2894, 2941, 2944	140, 311, 468, 637, 1012, 1016, 1068, 1072, 1110, 1174, 1375, 1461, 1469, 1538, 2894, 2941, 2944	140, 311, 468, 637, 1012, 1068, 1110, 1174, 1375, 1461, 1469, 1538, 2894, 2941, 2944, 1016
**3**			140, 311, 468, 637, 1012, 1016, 1068, 1072, 1110, 1174, 1375, 1461, 1538, 2894, 2941, 2944	140, 311, 468, 637, 1012, 1016, 1068, 1072, 1110, 1174, 1375, 1461, 1469, 1538, 2894, 2941, 2944	140, 311, 468, 637, 1110, 1375, 1461, 1469, 1538, 2894, 2944
**4**				311, 468, 637, 1012, 1016, 1068, 1110, 1174, 1375, 1461, 1469, 1538, 2894, 2941, 2944	140, 311, 468, 637, 1012, 1016, 1068, 1072, 1110, 1174, 1375, 1461, 1469, 1538, 2894, 2941, 2944
**5**					140, 311, 468, 637, 1012, 1016, 1068, 1072, 1110, 1174, 1375, 1461, 1469, 1538, 2894, 2941, 2944

**Table 3:** Classification metrics.

	Precision	Recall	F1-Score	Quantity
**(1–3)**	0.97	0.97	0.97	33
**(4–6)**	0.97	0.97	0.97	31
**Accuracy**	**0.97**			64

## Data Availability

All data generated or analyzed during this study are included in this published article. Any additional data will be available upon request.
